# Development of DOTA-Rituximab to be Labeled with ^90^Y for Radioimmunotherapy of B-cell Non-Hodgkin Lymphoma

**Published:** 2017

**Authors:** Fariba Johari doha, Siyavash Rahmani, Pedram Rikhtechi, Samira Rasaneh, Zahra Sheikholislam, Soraya Shahhosseini

**Affiliations:** a *Radiation application research school, Nuclear Science and Technology research Institute (NSTRI), Tehran, Iran. *; b *PET/CT unit, Ferdous Nuclear Medicine Center, Dr Masih Daneshvari Hospital, Shahid Beheshti University of Medical Sciences, Tehran, Iran. *; c *Department of Pharmaceutical Chemistry and Radiopharmacy, School of Pharmacy, Shahid Behesti University of Medical Sciences, Tehran, Iran.*

**Keywords:** B-cell Non-Hodgkin Lymphoma, radioimmunotherapy, 90-Y, Rituximab, Immunoreactivity

## Abstract

NHL is the most common hematologic cancer in adults. Rituximab is the FDA approved treatment of relapsed or refractory low grade B-cell Non-Hodgkin Lymphoma (NHL). But patients eventually become resistant to rituximab. Since lymphocytes and lymphoma cells are highly radiosensitive, low grade NHL that has relapsed or refractory to standard therapy is treated by RIT in which a beta-emitting radionuclide coupled to anti-CD20 antibody. The association of beta emitter radionuclide to rituximab enhances its therapeutic efficacy. The cells which lack antigen or cells which cannot be reached due to poor vascularization and intratumoral pressure in a bulky tumor would be irradiated and killed by cross fire effect of beta emitter. ^90^Y, a pure high energy β-emitter with a half-life of 64 h, a maximum energy of 2.28 MeV, and maximum board of 11.3 mm in tissue is radionuclide of choice for radioimmunotherapy of outpatient administration.

In this study, rituximab was conjugated to DOTA and radiolabeled with ^90^YCl_3_. The stability, affinity, and immunoreactivity of radiolabeled antibody was determined in vitro and the conditions were optimized. Biodistribution studies were done in normal mice. The optimum conditions of conjugation and radiolabeling was 1-2 h at 37 °C and 1 h at 45 °C, respectively. Results showed approximately 4 DOTA molecules conjugated per antibody molecule. The purified antibody was stable and intact over 6 months stored at -20 °C. The result of immunoreactivity (≈70%), affinity (≈3 nM) and biodistribution in normal mice are acceptable.

## Introduction

One promising strategy for treating malignancies is targeted in situ radiotherapy. In this approach, monoclonal antibodies (Mabs) or peptides are used as targeting vehicles to selectively deliver radionuclides to cancer cells for in situ radiation therapy. This approach is known as Radioimmunotherapy (RIT) if Mabs that recognize tumor associated antigens are used as targeting vehicles. NHL is the most common hematologic cancer in adults. Rituximab, a chimeric IgG1 anti-CD20 Mab, is the first FDA approved agent for treatment of relapsed or refractory CD20-positive low grade non-Hodgkinʹs lymphoma (NHL). Rituximab targets the CD20 antigen on benign and malignant B-cells and is now used for B-cell malignancies and autoimmune conditions. CD20 is a 33-37 kDa un-glycosylated transmembrane phosphoprotein. CD20 is an effective target for treatment of malignant B-cells since it does not circulate as a free antigen in the plasma, specifically expressed on the surface of normal and malignant B-cells but not on stem cells or other healthy tissues, and is not shed or internalized after antibody binding (1-3). CD20 functions as a calcium channel important for regulating cell cycle progression and calcium homeostasis. Rituximab kills CD20-positive B-cells. Several mechanisms have been suggested for therapeutic efficacy of rituximab including, antibody-dependent cellular cytotoxicity (ADCC), complement-dependent cytotoxicity (CDC), and the induction of apoptosis. But patients eventually become resistant to rituximab (4-5). Since lymphocytes and lymphoma cells are highly radiosensitive, low grade NHL that has relapsed or refractory to standard therapy is treated by RIT in which a beta-emitting radionuclide coupled to anti-CD20 antibody. The association of beta emitter radionuclide to rituximab enhances its therapeutic efficacy. The cells which lack antigen or cannot be reached due to poor vascularization and intratumoral pressure in a bulky tumor would be irradiated and killed by cross fire effect of beta emitter. ^90^Y is a pure, high energy β-emitter with a half-life of 64 h, a maximum energy of 2.28 MeV, and maximum board of 11.3 mm in tissue. High doses of antibody labeled 90-Y can be used for outpatient administration because of no gamma emission (1, 6-8). In this study, rituximab was conjugated to DOTA and radiolabeled with ^90^YCl_3_. The stability, affinity, and immunoreactivity of radiolabeled antibody was determined in vitro and the conditions were optimized. Biodistribution studies was done in normal mice.

## Materials and methods

Silica gel 60 F_254_ pre-coated aluminium sheets from Merck were used for TLC. The distribution of radioactivity on TLC was determined using a TLC Scanner Mini-Scan, MS.1000. This was equipped with flow count B-FC-1000 and gamma detector MS3200, (Bioscan, Washington, USA). A NaI well counter (Triathler multilabel tester, Hidex, Finland) and a dose calibrator (Atomlab 100, Biodex, NY) 

1= F.J.D and S.R contributed equally to this work.

 were used to measure low and high levels of radioactivity, respectively. Flow cytometric analysis were performed using a flow cytometer equipped with its accompanying software (FACSCalibur and CellQuestPro, respectively, Becton Dickinson). Size exclusion HPLC (SE-HPLC) analysis was performed using a Merck Hitachi, UV-Vis HPLC, column: TSK-Gel G3000SW_XL_, 7.8 mm ID, 30 cm L, 5 µM, mobile phase: PBS pH 7.4, flow rate: 0.8 mL/min, sample volume: 20 µL (20 µg). Raji cells, human Burkitt CD20 antigen positive lymphoma cells, Pasture Institute, Tehran, Iran. Anti-human IgG (FC specific)-FITC antibody produced in goat, affinity isolated antibody (Sigma-Aldrich, MO, USA). ^90^Ycl_3_ was provided from a ^90^Sr/^90^Y electrochemical generator (Pars-isotope, Tehran, Iran). P-SCN-Bz-DOTA was purchased from Macrocyclic, Dallas, TX, USA. 

*Purification of Rituximab for conjugation*

Chimeric anti-CD20 Rituximab as a Pharmaceutical sample (Zytux) from Aryogen Biopharma Co, Karaj, Iran, 100 mg/10 mL was purified and concentrated by centrifugation at 1500 g using Centricon filter (Sartorius, MWCO 30,000). The solution was washed five times with sodium carbonate buffer pH 8.6 (Na_2_CO_3_ 2 mM, NaHCO_3_ 48 mM, NaCl 150 mM). 

*Determination of concentration, purity, and integrity of purified Rituximab*

The concentration, purity, and integrity of antibody were determined based on following methods. 

The concentration was determined by UV absorbance at 280 nm using an extinction coefficient of 1.4 and Bradford assay (1976) using BSA as a standard (9). The purity was determined by size exclusion chromatography (SE HPLC). The integrity was determined using SDS-PAGE at reducing and non-reducing conditions. Purified antibody was aliquoted and stored at -20 °C for further experiments. 

*Conjugation of p-SCN-Bz-DOTA to Rituximab*

Aliquots of p-SCN-Bz-DOTA (Macrocyclics, Dallas, TX, USA), 2 mg/mL in sodium carbonate buffer pH 8.6, was mixed slowly with aliquots of rituximab solution (20 mg/mL in carbonate buffer pH 8.6) molar ratio Mab:DOTA 1:20 or 1:10. Reaction mixture was incubated at room temperature overnight or 37 °C 1-2 h (10-12). The progress of reaction was checked by SE HPLC. The coupling reaction was terminated by centrifugation at 1500 g using Centricone filter (Sartorius, MWCO 30,000) to remove excess DOTA and exchange buffer to 0.25 M ammonium acetate buffer, pH 7. The concentration and integrity of Rituximab-DOTA were determined by UV at 280 nm, SE HPLC, and SDS-PAGE. The DOTA-rituximab was aliquoted and stored at -20 °C for further experiments. 

**Table 1:** Biodistribution of ^90^Y-DOTA-Rituximab in normal mice

**Tissue**	**4 h**	**24 h**	**72 h**
	**mean±SD (n=3)**		
**Blood**	32.78 ± 0.28	10.5 ± 1.34	2.96 ± 0.09
**Lungs**	34.69 ± 1.21	8.92 ± 0.13	4.91 ± 0.9
**Heart**	21.5 ± 2.07	5.71 ± 1.23	3.78 ± 0.34
**Liver**	25.32 ± 1.16	19.13 ± 3.3	14.13 ± 5.9
**Spleen**	28.67 ± 3.07	9.34 ± 1.49	18.76 ± 3
**Stomach**	2.40 ± 0.28	1.71 ± 0.6	0.62 ± 0.13
**Small intestine**	3.40 ± 0.27	1.85 ± 0.2	0.97 ± 0.3
**Large intestine**	4.51 ± 0.91	3.35 ± 0.9	2.12 ± 0.3
**Kidneys**	17.73 ± 0.87	8.2 ± 1.9	5.3 ± 0.58
**Bone**	6.01 ± 0.07	5.13 ± 0.17	4.5 ± 0.5

**Figure 1.**
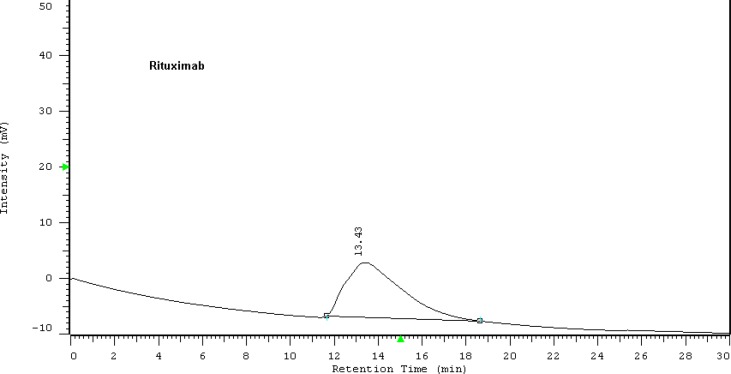
HPLC chromatogram of Zytux solution

**Figure 2:**
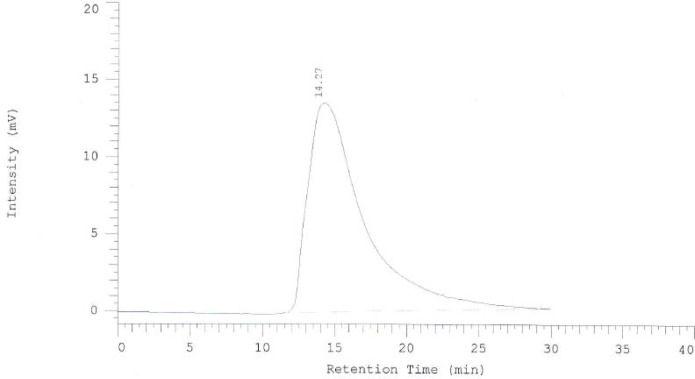
HPLC chromatogram of purified Rituximab

**Figure 3:**
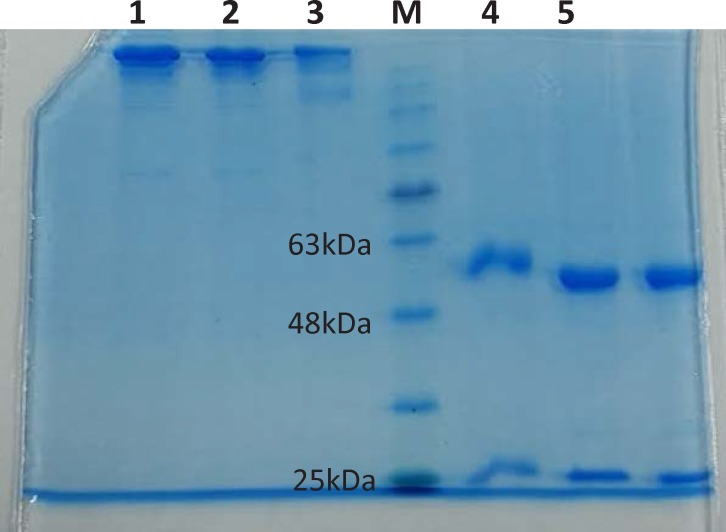
SDS-PAGE of Rituximab in reducing and non-reducing conditions.

**Non-reducing conditions**: **1**: Zytex, **2**: purified Rituximab, **3**: DOTA-Rituximab

**Reducing conditions**: **4**: DOTA-Rituximab, **5**: purifies Rituximab, **6**: Zytex

**M: **Molecular marker

**Figure 4:**
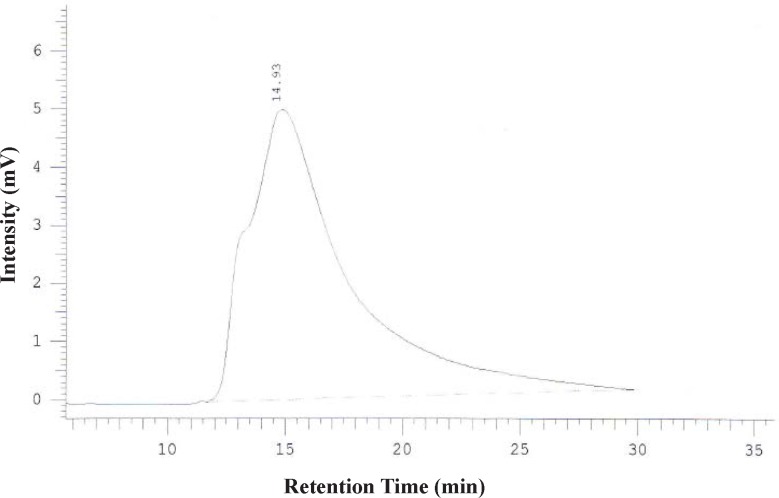
HPLC chromatogram of DOTA-Rituximab after purification using centricone

**Figure 5.**
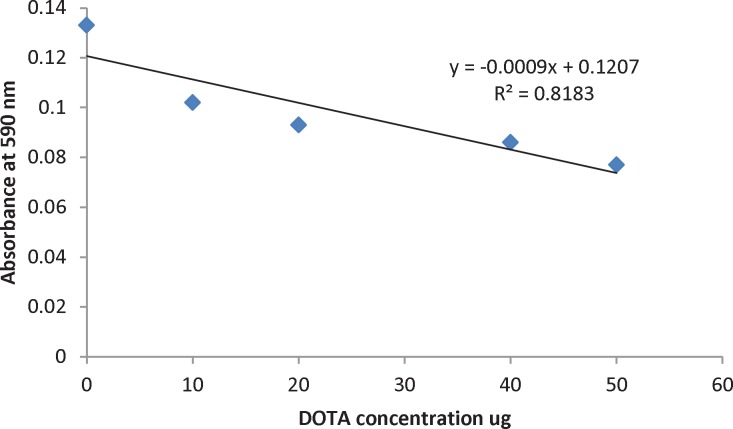
Calibration curve: Absorbance of Pb (II)-AA (III) complex at 590 nm upon adding DOTA ligand.

**Figure 6:**
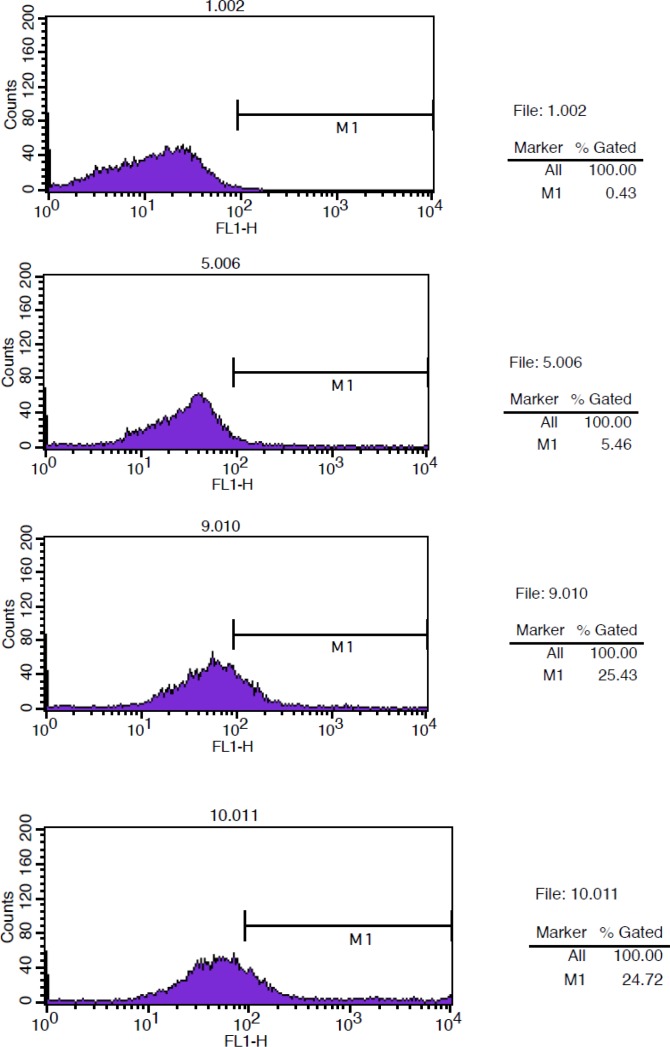
Flow Cytometry studies for determination immunoreactivity of DOTA-conjugated antibody. From up to down. Background: Cells + rituximab. Negative control: Cells + Herceptin + anti-human IgG (FC specific)-FITC.

Positive control: Cells + rituximab + anti-human IgG (FC specific)-FITC. Sample test: Cells + DOTA-rituximab + anti-human IgG (FC specific)-FITC

**Figure 7:**
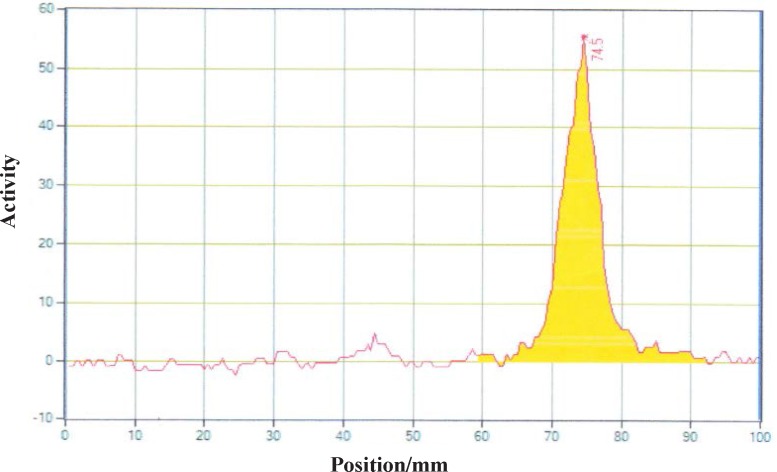
Radiochromatogram of ^90^YCl_3_ using TLC-SG, 0.1 M EDTA, 50 mM ammonium acetate pH7, R_f_^90^YCl_3_=0.75

**Figure 8:**
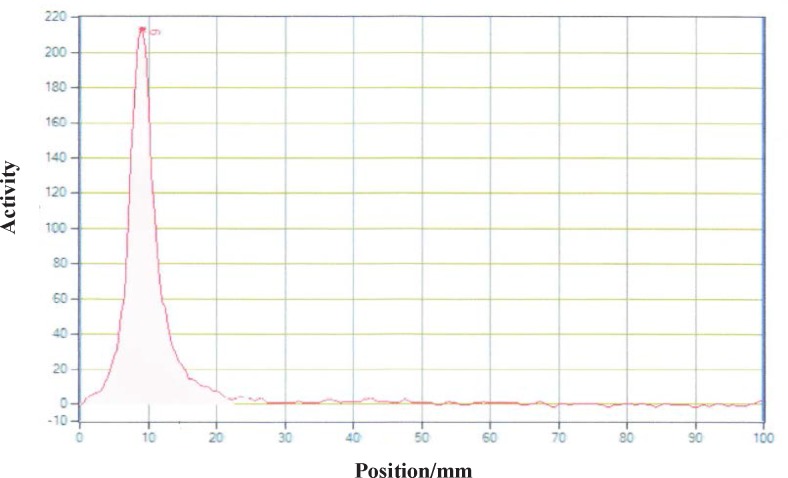
Radiochromatogram of ^90^Y-DOTA-Rituximab using TLC-SG, 0.1 M EDTA, 50 mM ammonium acetate pH7, R_f_^90^Y-DOTA-Rituximab=0.1

**Figure 9.**
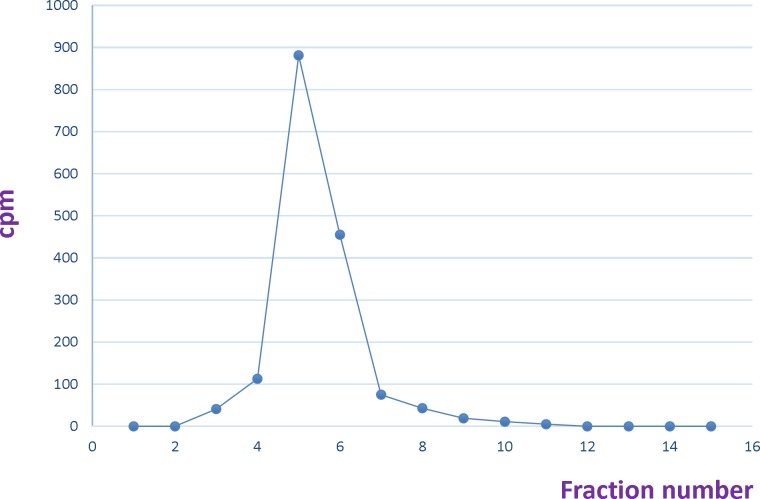
Size exclusion purification of ^90^Y-DOTA-Rituximab using PD-10 column. Fraction number 4 was considered as radiolabeled antibody

**Figure 10:**
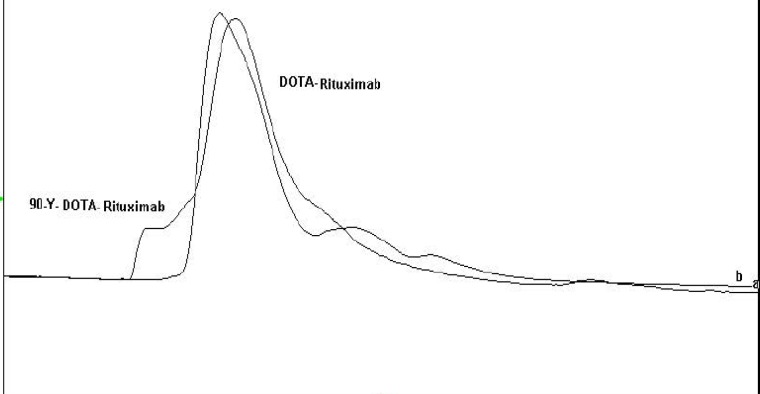
Overlapping HPLC chromatograms of ^90^Y-DOTA-Rituximab and DOTA-Rituximab.

**Figure 11:**
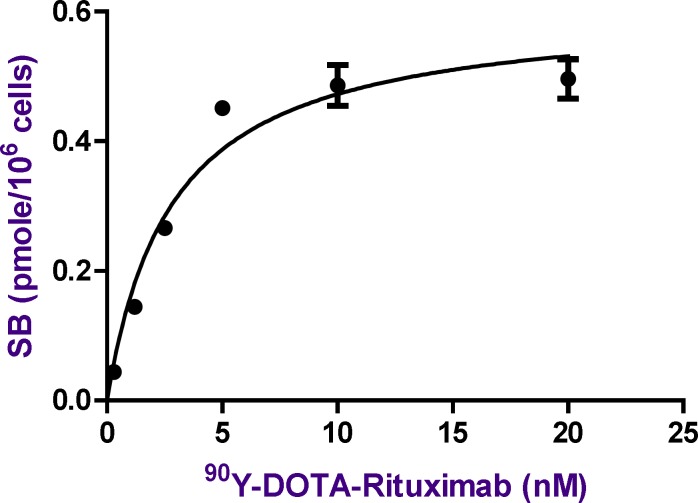
The Saturation curve for the binding of increasing concentrations of ^90^Y-DOTA-Rituximab in Raji cells. The amount of radioactivity bound to the cells, measured in cpm by gamma counter has been converted to nmol of ^90^Y-DOTA-Rituximab per cell in the incubation mixture. The values shown are the Mean±SD of three independent determinations

**Figure 12:**
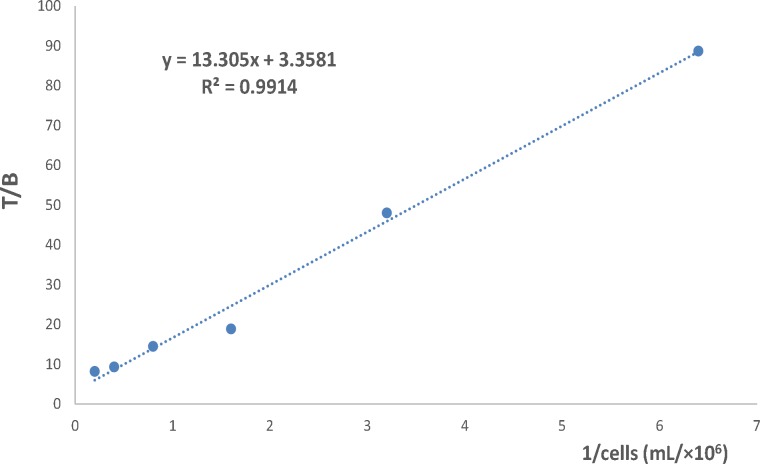
The immunoreactive fraction of ^90^Y-DOTA-Rituximab

**Figure 13:**
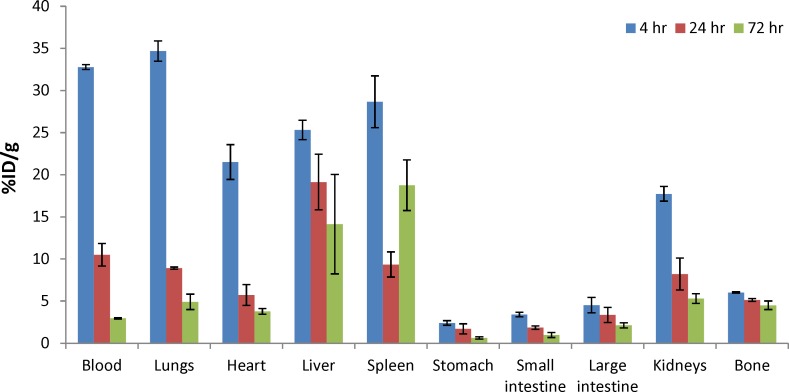
Biodistribution study of ^90^Y-DOTA-Rituximab in normal mice at 4, 24, and 48 hr post injection (n=3). Radioactivity is shown in terms of %ID/g organ. The values shown are the Mean±SD of three independent determinations

*Determination the number of DOTA conjugated per Rituximab molecule*

A complex was prepared of Arsenazo (III) [AA(III), Sigma-Aldrich] and Pb (II) (Atomic absorption standard solution, 1000 ppm Pb (II) in HNO3, Merck) in 0.15 M ammonium acetate buffer, pH 7 [10 µM AA(III) and 2 µM Pb (II)]. The complex was stored in the dark at 4 °C. 

A stock solution of DOTA (2 mg/mL in bicarbonate buffer) was prepared. For calibration curve, 850 µL AA (III)-Pb (II) complex, 50 µL NaCl 1 mg/mL, 0-25 µL DOTA solution, and 125-150 µL of 0.15 M ammonium acetate buffer, pH7 were incubated in the dark at room temperature for 20 min. The volume of each sample was 1050 µL. Absorbance of samples was read at 590 nm. Calibration curve was plotted from absorbance at 590 nm versus DOTA concentration.

To determine the ratio of DOTA to antibody, 850 µL AA (III)-Pb (II) complex, 50 µL NaCl 1 mg/mL, 100 µL DOTA-rituximab, and 50 µL of 0.15 M ammonium acetate buffer, pH7 were incubated in the dark at room temperature for 20 min (13-14). 

*Immunoreactivity of the DOTA-rituximab*

The immunoreactivity of the DOTA-rituximab was determined by flow cytometry. The binding of DOTA-rituximab and unconjugated rituximab to CD20 positive Raji cells was compared. Raji cells were grown in RPMI-1640 medium supplemented 10% heat inactivated fetal bovine serum (FBS). Cells were centrifuged, washed 2X with PBS (1% BSA), re-suspended in PBS (1% BSA), and aliquoted in Eppendorf tubes pre-coated with 1%BSA in PBS (1×10^6^ cells/tube). Cells were incubated at 4 ºC for 2 h with 100 µg rituximab (positive control), 100 µg Herceptin (negative control) and 100 µg DOTA-rituximab (test solution). Cells were washed twice with PBS (1% BSA), re-suspended in 1 mL PBS (1% BSA) and incubated with 100 µL/well anti-human IgG (FC specific)-FITC (Sigma-Aldrich) diluted 1:100 in PBS (1% BSA) in the dark at 4 ºC for 30 min. Cells were washed twice with PBS (1% BSA), re-suspended in 1 mL PBS (1% BSA) and analyzed with FACSCalibur (Becton Dickinson) using cellquest software. Raji cells and Raji cells with rituximab were used as background for autofluorescense. 

*Stability studies of DOTA-Rituximab*

The integrity of DOTA-Rituximab stored at -20 °C in 0.25 M ammonium acetate buffer, pH 7 was determined by SE HPLC and SDS-PAGE at 1, 3, and 6 months after preparation. Each aliquot was labelled with ^90^YCl_3_ and radiochemical purity was determined by radio TLC. 

^90^ *Y labeling of DOTA-rituximab*

90-Yttrium as yttrium chloride in 0.05 N HCl with Radioactivity concentration of 45.45 mCi/mL (1.68 GBq/mL), and 2 ppm ^90^Sr from a electrochemical ^90^Sr/^90^Y generator (ITD: isotope technology Dresden) was provided by Pars Isotope Co, Tehran, Iran. 

In the first step, the pH of Yttrium chloride was adjusted to 5-5.5 by using ammonium acetate buffer 0.5 M pH 7. Different amounts of DOTA-rituximab (10 µg-1 mg) was incubated with 1 mCi ^90^YCl_3_, pH5.5. The experiment was done at 37, 40, 42, 45, and 50 °C. The progress of reaction was checked by Radio-TLC. The final volume of reaction mixture was 0.5 mL using ammonium acetate buffer, pH7. The reaction mixture was purified by disposable PD-10 desalting column Sephadex G-25, (GE Healthcare life sciences) with PBS contains 2% BSA, pH7.4 as eluent. 15 fractions each 0.75 mL were collected and analyzed by Radio-TLC, TLC-SG as stationary phase and 0.1 M ammonium acetate pH 7, 50 mM EDTA as mobile phase. The fractions contained radiolabeled antibody (fractions 4-6) pulled together and analyzed by SE HPLC UV. 0.75 mL eluate fractions were collected using a fraction collector. The activity in each fraction was measured. A graph of activity against number of fractions was obtained (7, 10, 14-15). 

*Stability studies of* ^90^ *Y-DOTA-Rituximab in human plasma*

The stability of ^90^Y-DOTA-Rituximab was studied in human plasma. Briefly, 50 µCi (1 µCi/ µL) of labeled antibody was added to 450 µL of fresh human plasma and incubated for 4, 24, 48, and 72 h at 37 °C. At different time points, aliquots of the sample were removed and analysed by Radio-TLC. 

*Stability studies of* ^90^ *Y-DOTA-Rituximab in PBS*

The stability of ^90^Y-DOTA-Rituximab was studied in PBS. Briefly, purified fraction of radiolabelled antibody in PBS was kept at at 4 °C and room temperature. At different time points (4, 24, 48, and 72 h), aliquots of the sample were removed and analysed by Radio-TLC. 

*Saturation binding studies of* ^90^ *Y-DOTA-Rituximab*

A binding assay was performed in triplicate in the presence of increasing amounts of radiolabeled rituximab using Raji cells. 500 L of cell suspension in PBS pH7.4 (1×10^6^ cells) was added to 1 mL Eppendorf tubes (pre-incubated with 1% BSA in PBS at 4 °C, at least 30 min before experiment) and incubated with increasing concentrations of radiolabeled antibody (0.03-30 nM, specific activity 300 Ci/mmole) for 2 h at 4 °C with continuous rotation. At the end of incubation times, the mixture was centrifuged (1500 g, 10 min), the cell pellet was washed with cold 1% BSA in PBS (3X) and the radioactivity of pellet was measured as total binding (TB). For each radiolabeled rituximab concentration, nonspecific binding (NSB) was determined by incubation of cells with excess amount of unlabeled rituximab (100X of maximum concentration of radiolabeled antibody) (16-22). 

*Immunoreactivity of* ^90^ *Y-DOTA-Rituximab*

The immunoreactive fraction of ^90^Y-DOTA-Rituximab was determined based on method of Lindmo *et al.* (22-24). The study was performed in Eppendorf tubes (pre-incubated with 1% BSA in PBS at 4 °C, at least 30 min before experiment). A series of increasing concentration of cells (5×10^6^-0.156×10^6^) were incubated with 200 fold dilution of the saturation concentration of ^90^Y-DOTA-Rituximab for 2 h at 4 °C with continuous rotation. For each cell concentration, non-specific binding was determined by incubation of cells with 100 µg of cold antibody 30 min before adding radiolabeled antibody. At the end of incubation times, the mixture was centrifuged (1500 g, 10 min), the cell pellet was washed with cold 1% BSA in PBS (3X) and the radioactivity of pellet was measured. The immunoreactive fraction was determined using a double reciprocal of the number of total binding (TB) over specific binding (SB) counts against the reciprocal of the number of cells. The reciprocal of the Y-intercept equals the immunoreactive fraction (22-24). 

*Biodistribution studies in normal Balb/C mice*

Balb/C adult mice (6-8 week old) were used and obtained from the breeding facility of the Department of Pharmacology and Toxicology, School of Pharmacy, Shahid Beheshti University of Medical Sciences. All animal studies were conducted in accordance with the guidelines established by the Shahid Beheshti University of Medical Sciences. 100 µCi (50 µg) of radiolabeled antibody (1 µCi/1µL in saline, specific activity: 2 µCi/µg 74 MBq/mg) was injected via the tail vein of normal mice. The animals were sacrificed at 4, 24, and 72 h post injection (n = 3 for each time point). Interested organs and tissues were separated, weighted, and counted. The results were reported as percentage of injected dose per gram of organ (%ID/g). 

## Results and Discussion

To promote the clinical application of radioimmunotherapy for the most important cancers in developing countries, an IAEA Coordinated Research Project (International Atomic Energy Agency-CRP) was developed. The aim of our team was to optimize techniques for radiolabeling of Rituximab with ^90^Y. Rituximab was purified from Zytux solution (Aryogen Biopharma Co, Karaj, Iran, 100 mg/10 mL) using Centricone filter with cut off 30 kDa. The small molecules such as additives which might interfere with conjugation and labeling were removed, buffer exchanged to carbonate buffer that is used for conjugation, and antibody was concentrated. The concentration of purified rituximab was determined by UV at 280 nm using an extinction coefficient of 1.4 and formula [C(mg/mL)=(A_280_×dilution factor)/1.4]. The final concentration was 20 mg/mL. Rituximab was purified with recovery efficiency ≈ 80%. The antibody was aliquoted and stored at -20 °C. The integrity and purity of antibody after purification was determined using SE HPLC (Figures 1-2) and SDS-PAGE (Figure 3). Figures 1 and 2 shows a typical HPLC UV of Zytux (R_t _= 13.43 min) and purified rituximab (R_t _= 14.27 min). In order to conjugate p-SCN-Bz-DOTA to rituximab, two different conditions, room temperature and 37 °C, were used. Antibody was incubated overnight at room temperature and 1-2 h at 37 °C in carbonate buffer with a ten to twenty fold excess of DOTA (molar ratio Mab:DOTA 1:10 or 1:20). At 1, 2, and 12 h incubation time, the reaction mixture was centrifuged using Centricone filter to remove excess DOTA, which was washed 8 times until the absorbance of filtrate was close to zero. The conjugated antibody was concentrate and exchanged buffer to ammonium acetate, which is used in labeling. Incubation of antibody with DOTA overnight at room temperature resulted in aggregation of antibody (≈100%). The best conditions were 1-2 h incubation at 37 °C, molar ratio Mab:DOTA 1:20 (Figure 4). As it seen from Figure 4 less than 5% of antibody aggregated. The concentration of rituximab was determined by UV at 280 nm using an extinction coefficient of 1.4. The integrity and purity of DOTA conjugated antibody after purification was determined using SE HPLC (R_t _= 14.93 min) (Figure 4) and SDS-PAGE (Figure 3). 

The final concentration was 20 mg/mL. The antibody was aliquoted and stored at -20 °C. The SDS-PAGE results showed the same pattern for Zytux (Rituximab commercial sample), Purified rituximab and DOTA conjugated rituximab in non-reducing conditions (one band at ≈ 150 kDa) and reducing conditions (two bands at ≈ 50 kDa and ≈ 25 kDa), which means conjugation has no effect on integrity of antibody. Purified rituximab and DOTA-rituximab stored at -20 °C were analyzed by SE HPLC and SDS-PAGE over 6 months. HPLC chromatogram and SDS-PAGE showed that purified rituximab and DOTA-rituximab were nearly 100% intact after 6 months (data not shown). 

The number of DOTA conjugated per antibody molecule was determined using a spectrophotometric method based on a complex between AA (III) and Pb (II). The absorbance of complex at 590 nm was decreased by adding known amount of DOTA which was used to plot the calibration curve (Figure 5). From the calibration curve the number of DOTA conjugated per antibody molecule is determined. The conjugation of twentyfold DOTA to rituximab resulted in a DOTA-rituximab conjugate with an approximate ratio of 4 DOTA molecules per antibody. 

The binding of (DOTA)_4_-rituximab was compared with unconjugated rituximab antibody on Raji cells. Cells were incubated with rituximab (+Ctr), Herceptin (-Ctr), and DOTA-rituximab. The fluorescence intensity was analyzed after incubation with anti-human IgG (FC specific)-FITC using FACSCalibur and cellquest programe. Raji cells and Raji cells with rituximab were used as background. The results showed that the immunoreactivity of (DOTA)_4_-rituximab is approximately the same as rituximab (Figure 6). 

Different amounts of (DOTA)_4_-rituximab (0.5-1) mg was labeled with 1 mCi ^90^YCl_3_. The pH of the reaction mixture was 5.5 using ammonium acetate buffer, pH7. The reaction was incubated at 45 °C. After 1 h incubation, an aliquot was taken and analyzed by TLC (TLC-SG, 50 mM EDTA, 0.1 M ammonium acetate pH7, R_f_^90^Y-DOTA-rituximab = 0.1, R_f_^90^YCl_3 _= 0.75) (Figures 7-8). To remove free ^90^YCl_3, _the reaction mixture was passed through PD-10 column (Figure 9). Fractions were collected and analyzed by Radio-TLC. Fractions (4-6), which contains radiolabeled antibody were analyzed by SE HPLC. Results of SE HPLC showed that less than 5% of DOTA-Rituximab aggregrated during radiolabeling (Figure 10). The optimum conditions of radiolabeling of DOTA-Rituximab with ^90^Y (radiochemical purity ≈100%) is 0.5 mg DOTA-Rituximab, 1 mCi ^90^YCl_3_, pH5.5, 1 h at 45 °C, which means no purification step is needed to remove excess ^90^YCl_3_ before administration to the patient. The stability of ^90^Y-DOTA-Rituximab was studied in human serum plasma and PBS. At different time points, aliquots of the sample were removed and analysed by Radio-TLC. Results showed radiolabelled antibody was stable in human serum plasma at 37°C and PBS at room temperature and 4 °C. No appreciable loss in radiochemical purity was observed over 72 h. The radiochemical purity was ≈100%. 

The affinity (K_d_) of radiolabeled antibody to CD20 and the maximum number of antibody molecules bound per cell was determined by saturation binding studies. All of experiments were done in triplicates. Raji cells were incubated with increasing amount of radiolabeled antibody at 4 °C for 2 h. After incubation time, cell bound radiolabeled antibody were separated from free by centrifuge. The activity of pellet was considered as TB (Raji cells+radiolabeled antibody) or NSB (Raji cells+radiolabeled antibody+excess cold antibody). The amount of specific binding (SB) was calculated by subtracting NSB from TB (TB-NSB=SB). TB and NSB were determined at various radiolabeled antibody concentration. A plot of SB against concentration of radiolabeled antibody was used for calculation of affinity (k_d_) and B_max_. The curve was fitted according to a sigmoidal dose response profile using GraphPad Prism (Figure 11). As the concentration of radiolabeled antibody increases, the amount of bound increases until a point is reached where no matter how much more radioligand is added, the amount bound does not increase further. As shown in Figure 11, the binding parameters of ^90^Y-DOTA-Rituximab were calculated from the saturation binding experiments. The affinity of ^90^Y-DOTA-Rituximab K_d_ (equilibrium dissociation constant) was calculated as 2.836 ± 0.4602 nM. The published K_d_ by Stopar (2005) is 2.9 nM. The maximum binding capacity of ^90^Y-DOTA-Rituximab B_max_ (receptor density) was calculated as 0.6061 ± 0,03096 pmol (91 ng) per 10^6^ cells, which indicates that approximately 3.6×10^5^ molecules of ^90^Y-DOTA-Rituximab can be bound per cell at saturation. The B_max_ will vary from cell line to cell line and depend upon culture conditions. 

Because the conjugation and radiolabeling procedures may damage the antibody, it has to be determined how much the radiolabeled antibody is able to bind to the relevant antigen. The immunoreactive fraction of ^90^Y-DOTA-Rituximab was determined using method of Lindmo, a binding assay based on Lineweaver-Burk analysis. The immunoreactive fraction is expressed as the amount of bound antibody relative to the total amount applied. A series of increasing concentration of cells (10×10^6^-0.156×10^6^) were incubated with 200 fold dilution of the saturation concentration of ^90^Y-DOTA-Rituximab for 2 h at 4 °C. Under this conditions, the antigen is excess and all the antibody that is able to bind to antigen should be bound. At the end of incubation times, the activity of cell pellet was counted. TB, NSB, and SB were determined. Total added (TA) is the count of radiolabeled antibody in absence of cells and cold antibody. Results are shown in Figure 12. The immunoreactive fraction was determined using a double inverse of total binding over specific binding (TB/SB) counts against the reciprocal of the number of cells (1/No cell). The reciprocal of the Y-intercept equals the immunoreactive fraction (r). The immunoreactivity of ^90^Y-DOTA-Rituximab was 70%, approximately 70% of ^90^Y-DOTA-Rituximab is bound to Raji cell CD20 positive. 

The biodistribution of radioactivity after IV injection of ^90^Y-DOTA-Rituximab in normal mice is summarized as a function of time in Table 1 and Figure 13. Based on results, the main routes of elimination were hepatobiliary and urinary. Liver uptake (25.32 ± 1.16, 19.13 ± 3.3, and 14.13 ± 5.9 at 4, 24, and 72 h, respectively) and kidney uptake (17.73 ± 0.87, 8.2 ± 1.9, and 5.3 ± 0.58 at 4, 24, and 72 h, respectively). The urine was not collected. Yittrium-90, as a free radionuclide is a bone seeking toxic radionuclide (25). The low bone uptake at all times (less than 6%) confirms the in vivo stability of ^90^Y-DOTA-Rituximab. The blood activity decreased over a 72 h period (from 33% at 4 h to 2.96% at 72 h). The uptake of radiolabeled antibody in the stomach was low (less than 3%).

## Conclusion

Based on the results of this study, DOTA is a bifunctional chelating agent, which forms stable complexes with 90-Yttrium *in-vitro* and *in-vivo*. Rituximab, anti-CD20 monoclonal antibody, was conjugated and radiolabeled with 90-Yttrium by a fast and simple method and purified by PD-10 column with ≈ 80% recovery efficiency. The complex was stable in human plasma at 37 °C and PBS at room temperature and 4 C over 72 h, which is appropriate to use it routinely. The affinity and immunoreactivity of radiolabeled rituximab was determined in binding assays. Based on the results, the conjugation and radiolabeling does not destroy the immunoreactivity (70%) and affinity (K_d _= 2.8 nM) of antibody to the CD20 antigen. 
